# Discovery of lipid metabolism-related diagnostic biomarkers and construction of diagnostic model in steroid-induced osteonecrosis of femoral head

**DOI:** 10.1515/med-2025-1145

**Published:** 2025-08-12

**Authors:** Bin Zuo, Zhanchao Wang, Hua Lu, Hao Shen

**Affiliations:** Department of Orthopedics, Xinhua Hospital Affiliated to Shanghai Jiaotong University School of Medicine, Shanghai, 200092, China; Department of Orthopedics, Xinhua Hospital Affiliated to Shanghai Jiaotong University School of Medicine, Chongming Branch, Shanghai, 202150, China

**Keywords:** steroid-induced osteonecrosis of femoral head, lipid metabolism, diagnostic model, immune cells

## Abstract

This study aimed to identify candidate diagnostic biomarkers for steroid-induced osteonecrosis of the femoral head (SONFH). Two datasets were downloaded from the Gene Expression Omnibus for analyses of differentially expressed genes (DEGs) and lipid scores in SONFH and control groups and a weighted gene co-expression network analysis. Overlap between genes in the disease-related module, DEGs, and lipid metabolism-related genes was evaluated to obtain lipid metabolism-related DEGs. A diagnostic model was constructed and evaluated using a nomogram analysis and calibration curves. Correlations between immune cells and lipid metabolism-related DEGs in the model were evaluated. In total, 1,724 DEGs were screened between the SONFH and control groups. Seven SONFH-related modules and 18 lipid metabolism-related DEGs were identified. A total of six optimized genes, *CDK8*, *CHPT1*, *DPEP3*, *ORMDL3*, *PIP5K1B*, and *PPT2*, were obtained. A model based on these genes had good diagnostic performance for SONFH in GSE123568 and GSE74089. The six genes were significantly related to two immune cell subsets, myeloid dendritic cell activated and neutrophil. These six genes may be novel biomarkers for SONFH and the combination is a potential diagnostic signature.

## 1 Introduction

Nontraumatic osteonecrosis of the femoral head (NONFH) is a common debilitating orthopedic disease with high disability rate [1]. A large-scale epidemiological study conducted in China estimated that the cumulative number of patients with NONFH has reached 8.12 million [2]. The pain and restricted hip joint mobility resulting from femoral head necrosis can significantly impair the quality of life of affected individuals [3]. Steroid hormones are the primary cause of NONFH [4]. Hormones can lead to an increase in adipocytes in the femoral head and accumulation in the medullary cavity, which increases the pressure in the medullary cavity and compresses the surrounding blood vessels, eventually leading to steroid-induced ONFH (SONFH) [5]. SONFH is a debilitating orthopedic condition characterized by the progressive deterioration of the hip joint in individuals aged 20–50 years [6,7]. As the disease advances, the range of joint motion decreases gradually. When necrotic lesions exceed 30% in volume, the collapse rate of the femoral head can reach 80%, severely impacting quality of life [8]. However, SONFH is challenging to diagnose in its early stages and difficult to reverse in its advanced stages. Therefore, early diagnosis utilizing effective biomarkers and interventions are of great clinical significance.

Abnormal lipid metabolism is a primary pathological process in osteonecrosis [9]. Hormone drugs increase blood lipids, and the abnormal distribution of lipids can cause fat emboli in microvessels, leading to ischemic necrosis of the femoral head. In cases of SONFH, lipid accumulation within osteocytes causes these cells to enlarge, compressing the nucleus and compromising the integrity of the cell membrane, ultimately resulting in cell death and necrosis [10]. Ren et al. confirmed that glucocorticoid-induced ONFH is closely associated with lipid metabolism disorders induced by high-dose steroid administration *in vivo* [11,12]. Jiang et al. [13] found that the occurrence of SONFH could be inhibited by reducing the content of blood lipids, which could promote the growth of osteoblasts and inhibit adipocyte gene expression. Nozaki et al. [14] found that pravastatin can decrease the content of adipocytes within the femoral head of rats by regulating lipid metabolism, which in turn inhibits the occurrence of ONFH. Yeh et al. [15] confirmed that the expression of genes related to bone formation can be inhibited by hormones and increase the expression of lipid-related genes. However, the particular molecular mechanism by which lipid metabolism contributes to SONFH needs to be investigated.

In this study, the relationship between SONFH and lipid metabolism-related genes were studied. Genes that are significantly related to lipid metabolism and differentially expressed in SONFH were screened, and a diagnostic model based on these genes was constructed. The results provide a basis for diagnosis as well as potential therapeutic targets for SONFH.

## 2 Methods

### 2.1 Dataset and samples

GSE123568 and GSE74089 were downloaded on November 6, 2023 from the National Center for Biotechnology Information (NCBI) Gene Expression Omnibus (GEO) database [16] (http://www.ncbi.nlm.nih.gov/geo/). GSE123568 [17], used as a training dataset, contained 40 human blood serum samples from 30 patients with SONFH and 10 normal controls. The detection platform for GSE123568 was GPL15207 ([PrimeView] Affymetrix Human Gene Expression Array). GSE74089, used as the validation dataset, contained eight human synovial tissue samples from four patients with NFH and four normal controls (CTRL). The platform for GSE74089 was GPL13497 (Agilent-026652 Whole Human Genome Microarray 4x44K v2).

### 2.2 Screening of significantly differentially expressed genes (DEGs)

The limma package (version 3.34.7) (https://bioconductor.org/packages/release/bioc/html/limma.html) of R3.6.1 language [18] was used to screen DEGs between the control and SONFH groups in the GSE123568 dataset. False discovery rate (FDR) < 0.05 and |log_2_fold change (FC)| > 0.5 were selected as thresholds. Then, based on the DEGs, the pheatmap package (version 1.0.8) [19] (https://cran.r-project.org/package=pheatmap) in R3.6.1 was used for a bi-directional hierarchical clustering analysis of the expression values [20,21]. The results were visualized using a heatmap.

### 2.3 Assessment of lipid scores

Relevant genes involved in lipid metabolism pathways were downloaded from the Molecular Signatures Database (MSigDB) module of the Gene Set Enrichment Analysis (GSEA) database (http://software.broadinstitute.org/gsea/downloads.jsp) [22]. The lipid scores for samples in the SONFH and CTRL groups were evaluated using the GSVA package (version 1.36.3) in R (http://www.bioconductor.org/packages/release/bioc/html/GSVA.html) [23]. Subsequently, the distributions of lipid scores in the SONFH and CTRL groups were compared using the Kruskal–Wallis test. Furthermore, the ability of the lipid score to identify SONFH was assessed through the receiver operating characteristic (ROC) curve method using the pROC package (version 1.12.1) (https://cran.r-project.org/web/packages/pROC/index.html) in R 3.6.1 [24].

### 2.4 Identification of disease- and lipid metabolism-related DEGs

A weighed gene co-expression network analysis (WGCNA) is a bioinformatics approach for constructing co-expression networks to identify modules associated with diseases and screening important pathogenic mechanisms or potential therapeutic targets [25]. Modules related to SONFH were screened using the WGCNA package (version 1.61) in R3.6.1 [26] (https://cran.r-project.org/web/packages/WGCNA/index.html) based on DEGs. The thresholds for module partitions were as follows: module set containing at least 100 genes and cutheight = 0.995. The correlations between each module and the disease status and lipid score were calculated, and the modules that were significantly correlated with these parameters were retained.

Then, lipid metabolism-related genes obtained from the GSEA database, genes in the modules that were correlated with the disease status and lipid score, and DEGs identified through screening were compared, and the overlapping genes were selected as lipid metabolism-related DEGs. A functional enrichment analysis of lipid metabolism-related DEGs was conducted using the Gene Ontology (GO)-Biology Process (BP) and Kyoto Encyclopedia of Genes and Genomes (KEGG) databases based on DAVID version 6.8 [27,28] (https://DAVID.ncifcrf.gov/). A *p*-value of less than 0.05 was selected as the threshold for significant enrichment.

### 2.5 Construction of a diagnostic model based on lipid metabolism-related DEGs

Based on the expression levels of lipid metabolism-related DEGs, univariate logistic regression analyses were performed using the rms package version 6.3-0 [29] (https://cran.r-project.org/web/packages/rms/index.html) in R3.6.1, retaining only those genes with a *p*-value less than 0.05. The lipid metabolism-related DEGs identified through univariate logistic regression analyses were further refined using the LASSO algorithm in the lars package (version 1.2) [30] (https://cran.r-project.org/web/packages/lars/index.html) in the R 3.6.1 software to select the optimal lipid metabolism-related DEG combination. Subsequently, to assess the diagnostic potential of lipid metabolism-related DEGs in SONFH, three machine learning algorithms – support vector machine (SVM), decision tree, and random forest – were applied. The SVM model was constructed using the e1071 package in R, the decision tree model was developed using the “rpart” package, and the random forest model was created using the “randomForest” package. The performance of each model was then evaluated using the ROC curve method in the R 3.6.1 pROC package (version 1.12.1), separately assessing the efficacy of model using both the GSE123568 training dataset and GSE74089 validation dataset. Finally, a nomogram model and calibration curves were constructed using the rms package (version 5.1-2) (https://cran.r-project.org/web/packages/rms/index.html) [31] in R3.6.1.

### 2.6 Immune correlation analysis

The samples in the GSE123568 dataset were used to evaluate the proportions of immune cell types via CIBERSORT (https://CIBERSORT.stanford.edu/index.php) [32]. Differences in immune cell frequencies between SONFH and CTRL were evaluated using the Kruskal–Wallis test in R3.6.1. Finally, correlations between the distribution of immune cells and lipid metabolism-related DEGs in the model were evaluated.

### 2.7 Cell culture and treatment

The MC3T3-E1 cell line was obtained from the Institute of Cell Bank for Biological Sciences (Shanghai, China). These cells were maintained in Dulbecco’s Modified Eagle Medium (Invitrogen, Carlsbad, CA, USA) supplemented with 10% fetal bovine serum, and incubated at 37°C in a 5% CO_2_ atmosphere. To simulate steroid-induced femoral head necrosis at the cellular level, MC3T3-E1 cells were exposed to various concentrations of dexamethasone (DXM) (1, 10, 100, 200, and 300 μM) for 24 h. MC3T3-E1 cells not treated with DXM served as the control group.

### 2.8 Cell viability

The Cell Counting Kit-8 (CCK-8; Dojindo, Tokyo, Japan) was used to evaluate the viability of MC3T3-E1 cells, both untreated and treated with DXM. Cells were seeded at a density of 5,000 cells per well in 96-well plates and incubated at 37°C with 5% CO_2_ for 72 h. Afterward, 10 μL of CCK-8 solution was added to each well and incubated for 2 h. Cell viability was measured at 450 nm using a microplate reader (BioTek, Winooski, VT, USA).

### 2.9 Quantitative real-time polymerase chain reaction (qRT-PCR)

Total RNA was isolated from the cell lines using RNAiso Plus (Takara, Beijing, China). The RNA was then reverse transcribed into complementary DNA using PrimeScript RT Master Mix (Takara). qRT-PCR was conducted using Power SYBR Green PCR Master Mix (Thermo, Waltham, MA, USA). The thermal cycling protocol included an initial denaturation at 95°C for 2 min, followed by 40 cycles of 95°C for 15 s and 60°C for 60 s. A dissociation step was performed at 95°C for 15 s, 60°C for 60 s, and 95°C for 15 s. Relative gene expression was determined using the 2^−ΔΔCt^ method, with *GAPDH* as the reference gene. Primer sequences are listed in Table 1.

**Table 1:** Primers sequences

Name of primer	Sequences (5′–3′)
CDK8-F	ACCTGTTTGAATACGAGGGCT
CDK8-R	TGCCGACATAGAGATCCCAGT
CHPT1-F	CACCGAAGAGGCACCATACTG
CHPT1-R	CCCTAAAGGGGAACAAGAGTTTG
DPEP3-F	GATGCGGAGTTTCCCACTCG
DPEP3-R	GGCACACATGCGGTGAATG
ORMDL3-F	AACACGCGGGTGATGAACAG
ORMDL3-R	AGGGACACTCACAAACGGGA
PIP5K1B-F	CTGGGAATAGGATACACAGTGGG
PIP5K1B-R	GCTGGGTAGGAACACACTTTC
PPT2-F	ACAGTGCTCGATCTCTTCGAT
PPT2-R	CAGCCTCTCGGAACCCTTG
OCN-F	CACTCCTCGCCCTATTGGC
OCN-R	CCCTCCTGCTTGGACACAAAG
RUNX2-F	TGGTTACTGTCATGGCGGGTA
RUNX2-R	TCTCAGATCGTTGAACCTTGCTA
COL1-F	GAGGGCCAAGACGAAGACATC
COL1-R	CAGATCACGTCATCGCACAAC
GAPDH-F	TGACAACTTTGGTATCGTGGAAGG
GAPDH-R	AGGCAGGGATGATGTTCTGGAGAG

### 2.10 Statistical analysis

Statistical analyses were performed using GraphPad Prism 9.0 (GraphPad Software), and the results are expressed as the mean ± standard deviation. Differences between two groups were evaluated using Student’s *t*-tests, while comparisons among multiple groups were conducted using one-way ANOVA. A *p*-value of less than 0.05 was considered significant.

## 3 Results

### 3.1 DEGs between SONFH and CTRL groups

**Figure 1:**
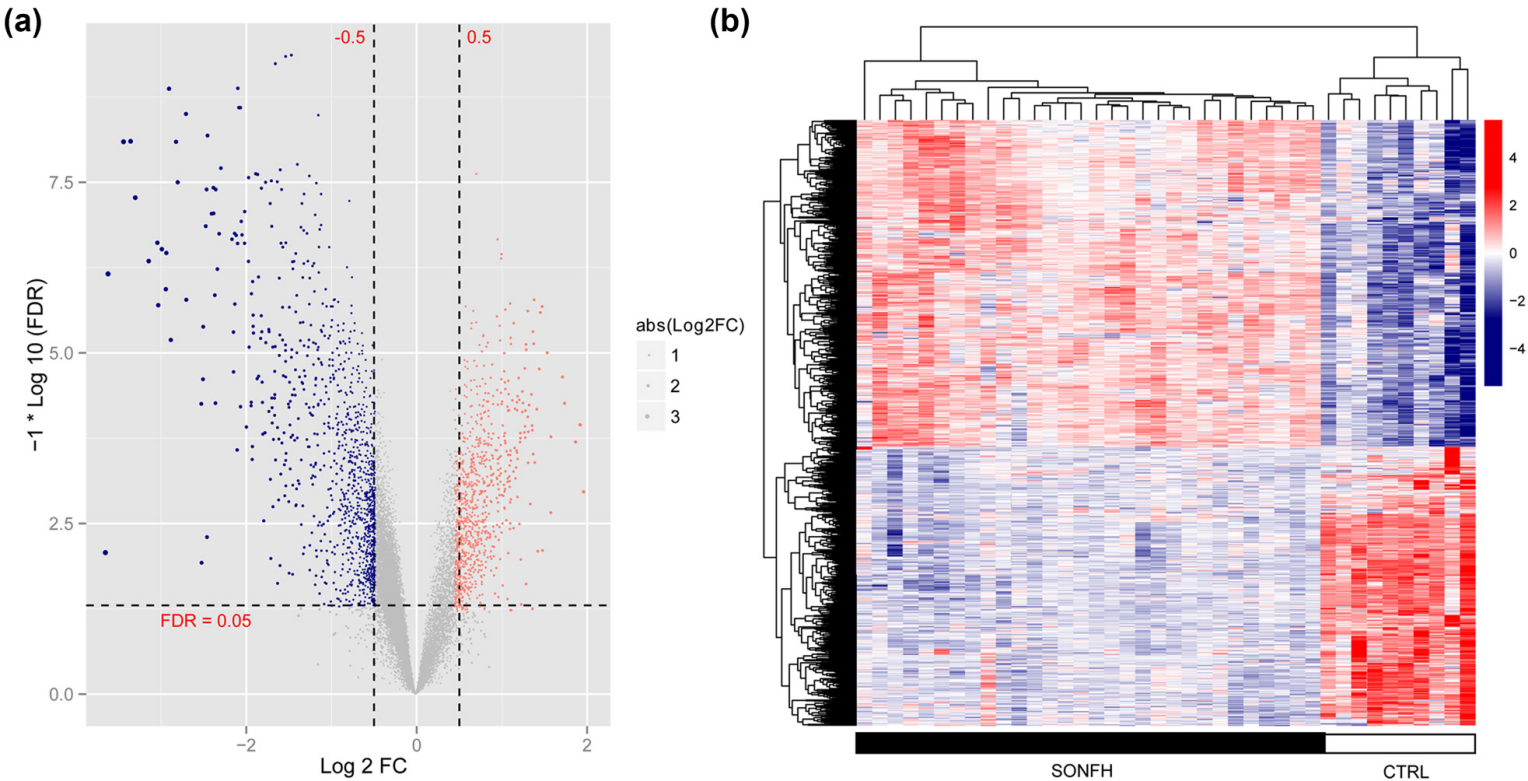
Volcano plot (a) and heat map (b) of DEGs between SONFH and CTRL groups. The gradient color change from blue to red represents expression changes, from downregulation to upregulation. FC: fold change. FDR: false discovery rate.

A total of 1,724 DEGs (948 downregulated and 776 upregulated) were screened between the SONFH and CTRL groups. A volcano diagram showing the gene expression differences between the two groups is shown in Figure 1a. A clustering heatmap based on the expression levels of DEGs indicated that genes with similar expression patterns were clustered together and could obviously distinguish SONFH and CTRL samples (Figure 1b).

### 3.2 Lipid scores

**Figure 2:**
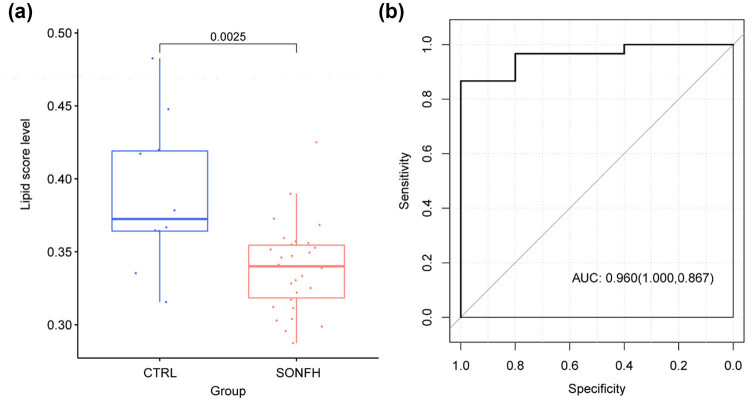
(a) Distribution of lipid scores in SONFH and CTRL groups. (b) ROC curve for the performance of the lipid score in identifying the sample type.

From the MSigDB of GSEA, 738 lipid metabolic pathway-related genes were obtained. The lipid score in the SONFH group was significantly lower than that in the CTRL group (*p* = 0.0025, Figure 2a). We examined the diagnostic value of the lipid score in SONFH. The results suggested that the lipid score effectively discriminates between the SONFH and CTRL groups with an AUC of 0.960, sensitivity of 100%, and specificity of 86.7% (Figure 2b).

### 3.3 Disease- and lipid metabolism-related DEGs

**Figure 3:**
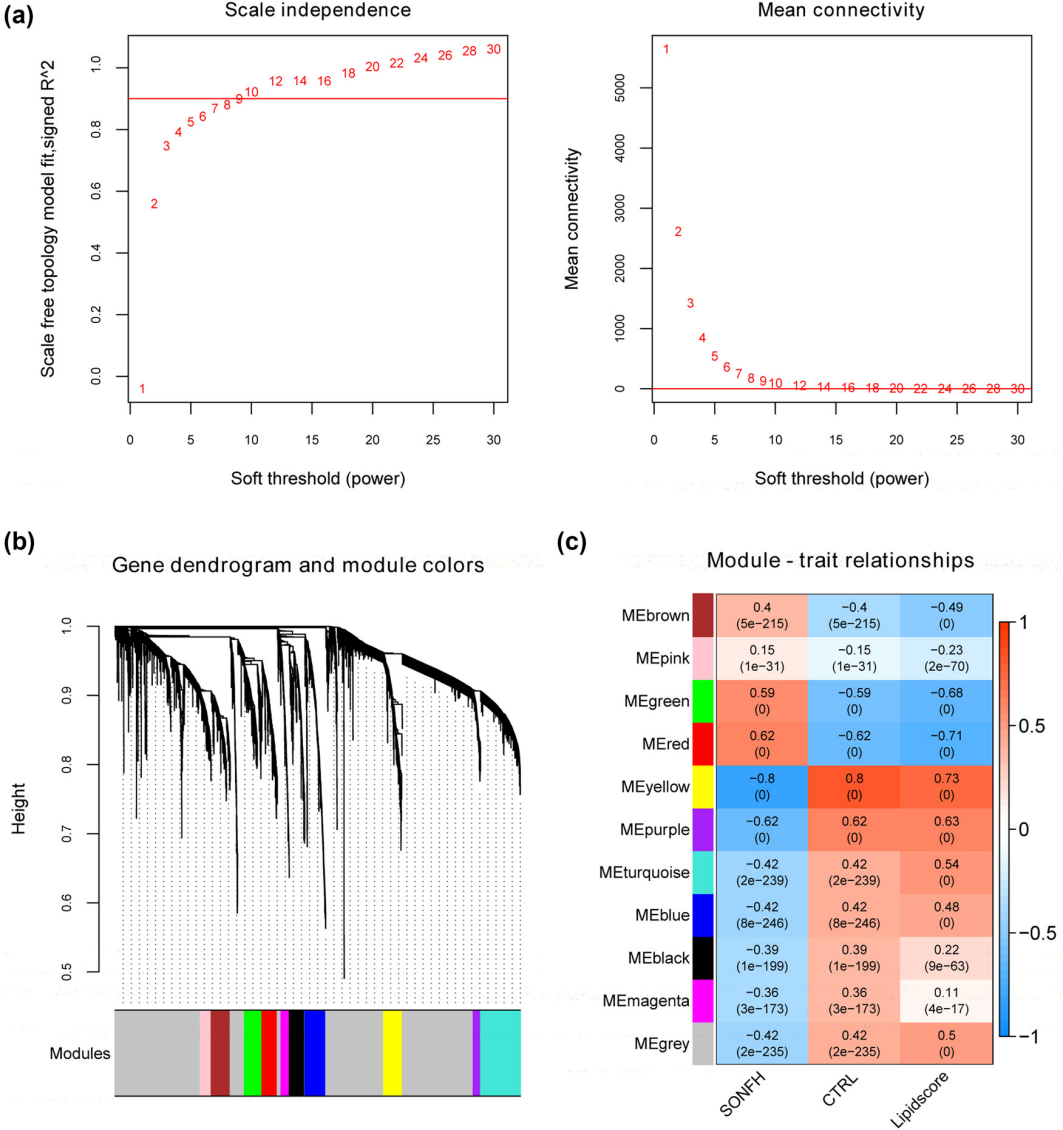
Weighted gene co-expression network analysis. (a) Selection of the soft threshold; (b) co-expression network of DEGs from GSE123568; (c) module–trait relationships: upper numbers for each module indicate the correlation coefficient, and numbers in brackets indicate the *p*-value.

A soft threshold power of 9 was chosen to create networks with a scale-free topology (*R* ^2^ reached 0.9; mean connectivity was equal to 1) (Figure 3a). In the dendrogram, the genes were clustered into 11 modules (Figure 3b). Correlations between these modules and the disease status as well as lipid scores are depicted in Figure 3c. Based on the correlation coefficients for relationships between each gene module and phenotypes (SONFH, CTRL, or lipid score), the yellow module exhibited the strongest correlations (cor  =  −0.80, *p*  =  0.00, Figure 3c).

**Figure 4:**
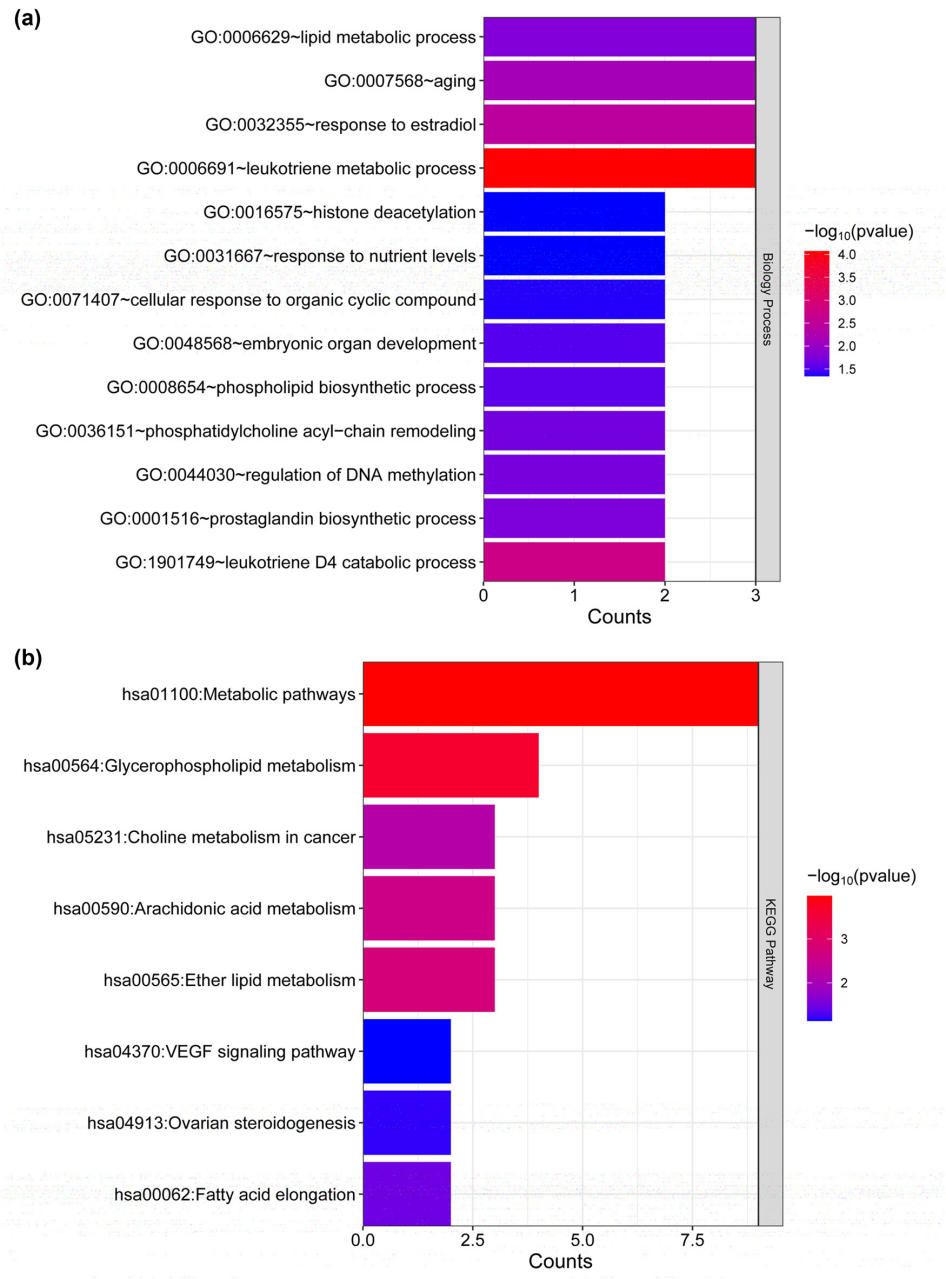
Functional pathway enrichment of lipid metabolism-related DEGs. (a) GO analysis of hub genes and (b) KEGG pathway analysis of hub genes. The horizontal axis represents the number of genes associated with the term, the vertical axis represents the term, and the color represents significance.

Among the 11 modules, the blue, brown, green, purple, red, turquoise, and yellow modules were significantly associated with both the disease state and lipid score (*p* > 0.3), encompassing a total of 1,929 genes. A Venn diagram analysis revealed 18 overlapping lipid metabolism-related DEGs among the lipid metabolism-related genes obtained from the GSEA database, genes within the modules correlated with the disease status and lipid score, and DEGs identified through screening. These 18 lipid metabolism-related DEGs were as follows: *ACSM3*, *SLC27A3*, *CDK8*, *PTGS2*, *THEM5*, *DPEP1*, *PCTP*, *PIP5K1B*, *CHPT1*, *ORMDL3*, *DPEP3*, *TMEM86B*, *PPT2*, *PTGR1*, *PLA2G4F*, *DPEP2*, *PISD*, and *GPAT3*. GO and KEGG functional analyses of the 18 lipid metabolism-related DEGs revealed 13 GO-BP terms, including lipid metabolic process, leukotriene metabolic process, response to estradiol, aging, and prostaglandin biosynthetic process, and 8 KEGG signaling pathways, including metabolic pathways, glycerophospholipid metabolism, ether lipid metabolism, VEGF signaling pathway, and arachidonic acid metabolism, were identified (Figure 4a and b).

### 3.4 Diagnostic model based on lipid metabolism-related DEGs

**Figure 5:**
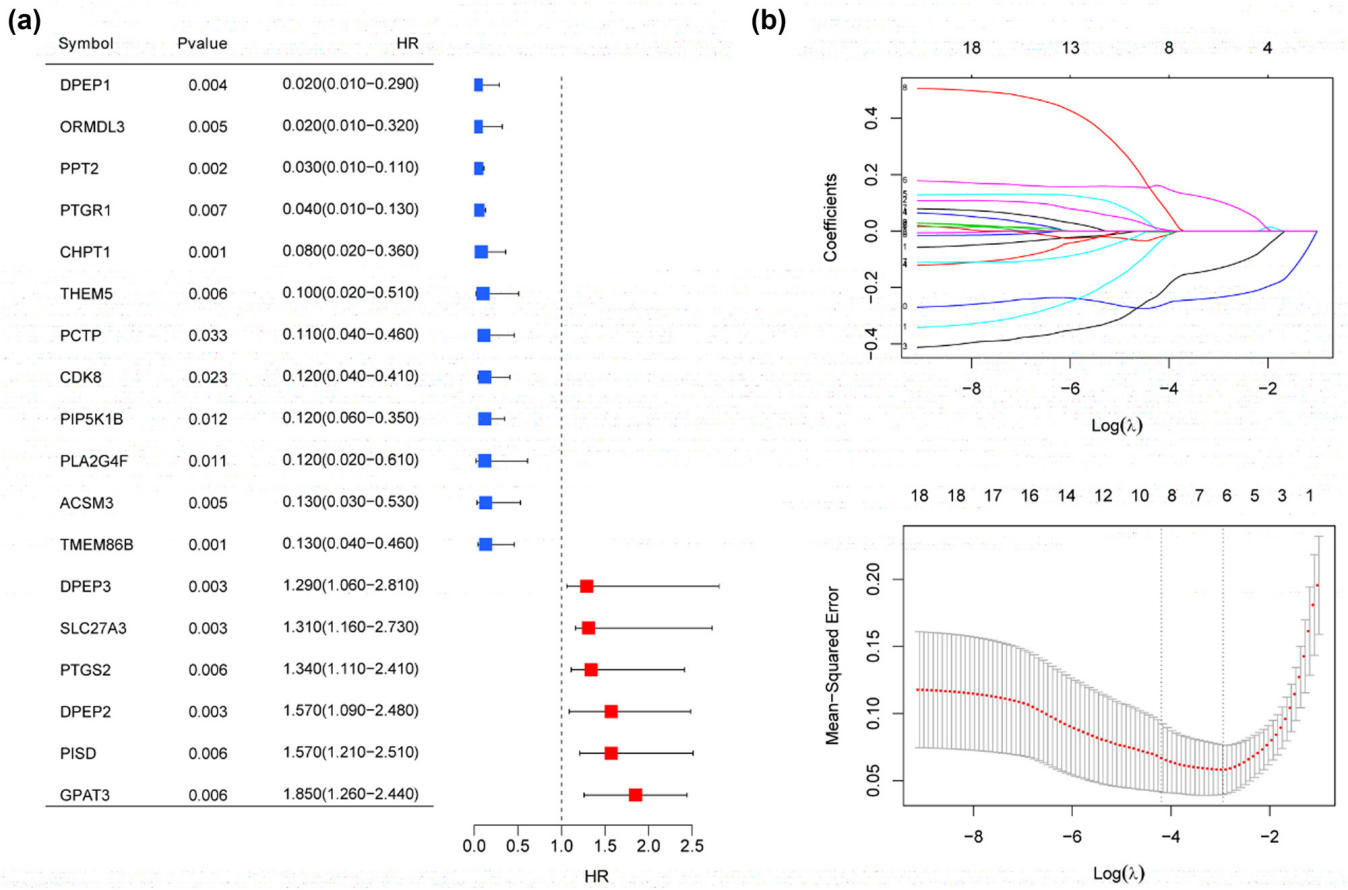
Univariate logistic regression analyses of lipid metabolism-related DEGs. (a) Screening of optimized genes associated with prognosis in GSE123568 by LASSO regression (b).

Univariate logistic regression analyses of the 18 lipid metabolism-related DEGs revealed significant associations with the disease state (*p* < 0.05) (Figure 5a). A LASSO analysis was performed to further screen these genes, resulting in the identification of six optimized lipid metabolism-related DEGs: *CDK8*, *CHPT1*, *DPEP3*, *ORMDL3*, *PIP5K1B*, and *PPT2* (Figure 5b).

**Figure 6:**
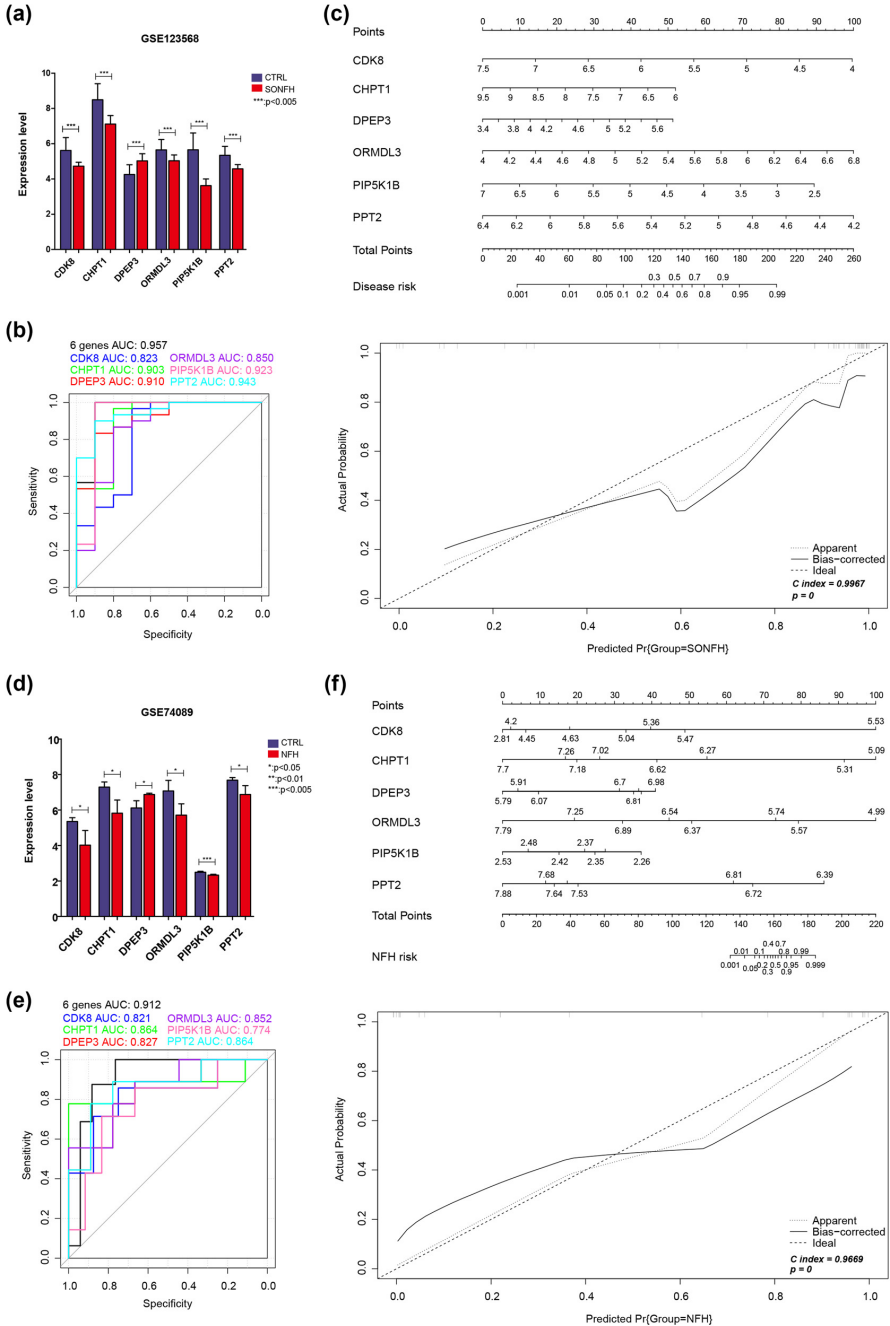
In GSE123568, the expression of six genes in the two groups (a), ROC curves and area under the curve for the candidate genes (b), and nomogram and calibration of the combination of six optimized genes (*CDK8*, *CHPT1*, *DPEP3*, *ORMDL3*, *PIP5K1B*, and *PPT2*) (c). In GSE74089, the expression of six genes in the two groups (d), ROC curves and area under the curve for the candidate genes (e), and nomogram and calibration of combination of six optimized genes (*CDK8*, *CHPT1*, *DPEP3*, *ORMDL3*, *PIP5K1B*, and *PPT2*) (f).

**Figure A1:**
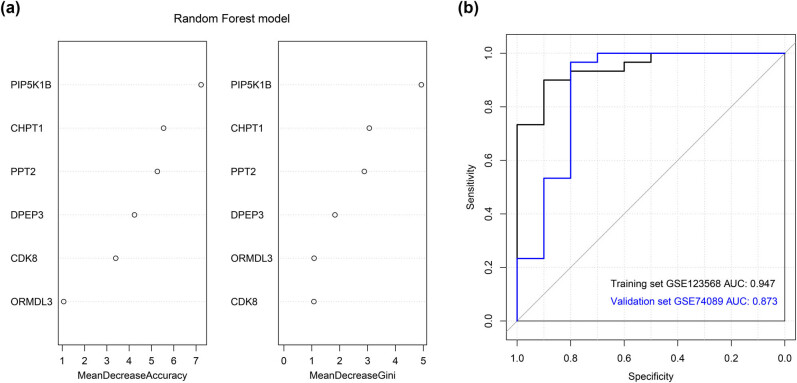
ROC results for the random forest model based on the six optimized genes for discrimination between the SONFH and CTRL groups.

**Figure A2:**
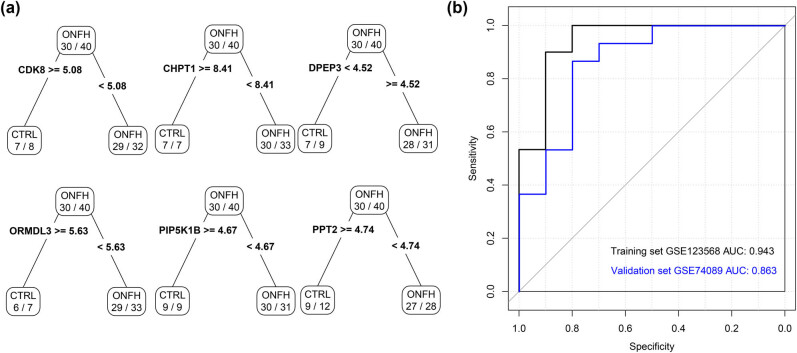
ROC results for the decision tree model based on the six optimized genes for discrimination between the SONFH and CTRL groups.

We further visualized the expression levels of these six optimized lipid metabolism-related DEGs in the GSE123568 and GSE74089 datasets. Compared with levels in the CTRL group, the expression levels of *CDK8*, *CHPT1*, *ORMDL3*, *PIP5K1B*, and *PPT2* were significantly lower in the SONFH group, while the expression level of *DPEP3* was significantly higher (*p* < 0.05, Figure 6a and d). The diagnostic value of the optimized lipid metabolism-related DEGs in SONFH was examined utilizing SVM, decision tree, and random forest models. The SVM model achieved AUC values of 0.957 for the GSE123568 dataset and 0.912 for the GSE74089 dataset (Figure 6b and e). The random forest model yielded AUC values of 0.947 for the GSE123568 dataset and 0.873 for the GSE74089 dataset (Figure A1). Similarly, the decision tree model recorded AUC values of 0.943 for the GSE123568 dataset and 0.863 for the GSE74089 dataset (Figure A2). These results indicate that all three models, developed based on the expression levels of six optimized lipid metabolism-related DEGs, demonstrate significant discriminatory ability between the SONFH and CTRL groups. Furthermore, the nomogram model and calibration curves for six genes across two databases are illustrated in Figure 6c and f. The concordance index (C-index) of the nomogram reached 0.9967 in GSE123568 and 0.9669 in GSE74089, indicating a high capacity for distinguishing between the SONFH and CTRL groups.

### 3.5 Validation of six optimized lipid metabolism-related DEGs

**Figure 7:**
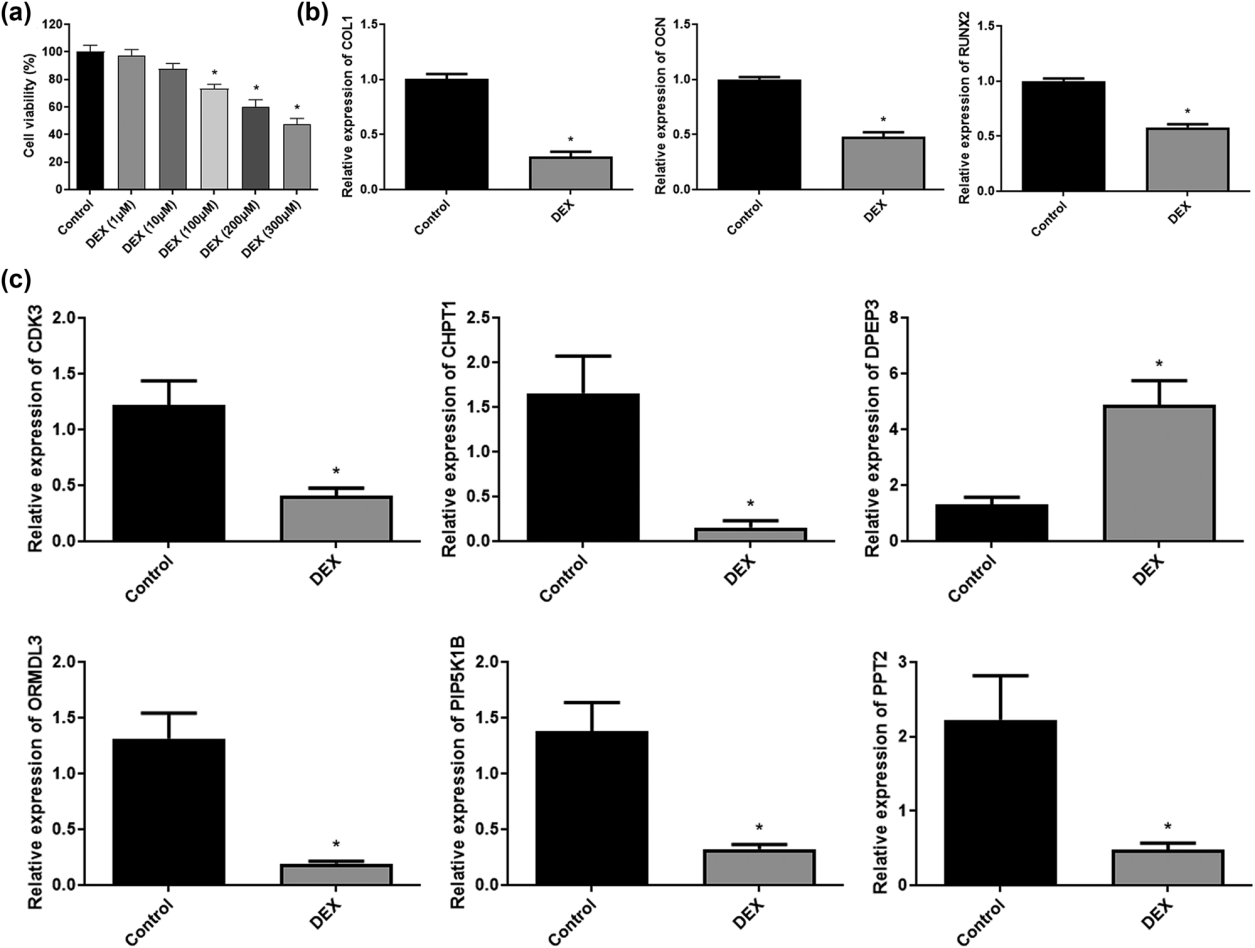
Validation of the six optimized genes. (a) CCK-8 assay was used to detect the viability of MC3T3-E1 cells and DXM-treated MC3T3-E1 cells. *p < 0.05, compared with the control. (b) qRT-PCR was used to detect the expression levels of *COL1*, *OCN*, and *RUNX2*. *p < 0.05, compared with the control. (c) qRT-PCR was used to detect the expression levels of *CDK8*, *CHPT1*, *DPEP3*, *ORMDL3*, *PIP5K1B*, and *PPT2*. *p < 0.05, compared with the control.

*In vitro* experiments were conducted to assess the expression of the six optimized DEGs related to lipid metabolism. The MC3T3-E1 cell line was treated with DEX to simulate SONFH. To determine the optimal DEX concentration for induction, cell viability was evaluated using CCK-8 assays after treatment with various DEX concentrations. After 24 h of treatment with 300 μM DEX, cell viability decreased by over 50% (Figure 7a). Consequently, 200 μM DEX was selected for further experiments. Following DEX treatment, mRNA expression analyses revealed that the osteogenesis-related genes *COL1*, *OCN*, and *RUNX2* were downregulated, indicating impaired osteogenic differentiation (*p* < 0.05, Figure 7b). Subsequently, qRT-PCR was used to analyze the mRNA expression levels of the six optimized lipid metabolism-related DEGs in both control and DEX-treated groups. The DEX group exhibited significantly lower levels of *CDK8*, *CHPT1*, *ORMDL3*, *PIP5K1B*, and *PPT2* than those in the control group, while *DPEP3* expression was significantly increased (*p* < 0.05, Figure 7c).

### 3.6 Relationships between candidate genes and differential immune cells

**Figure 8:**
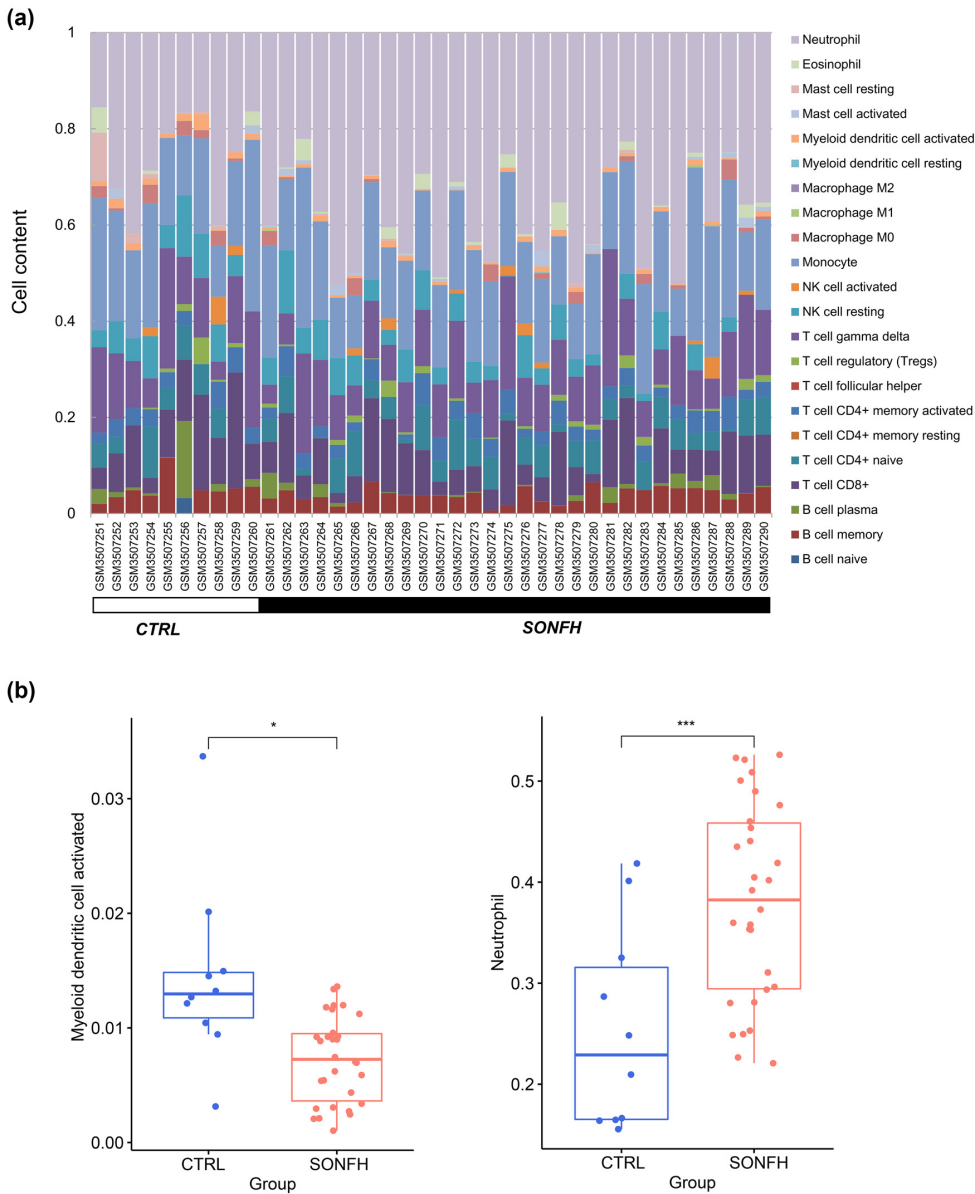
(a) Distribution of immune cells in two samples. (b) Two distinctly distributed immune cells (myeloid dendritic cell activated and neutrophil).

**Figure 9:**
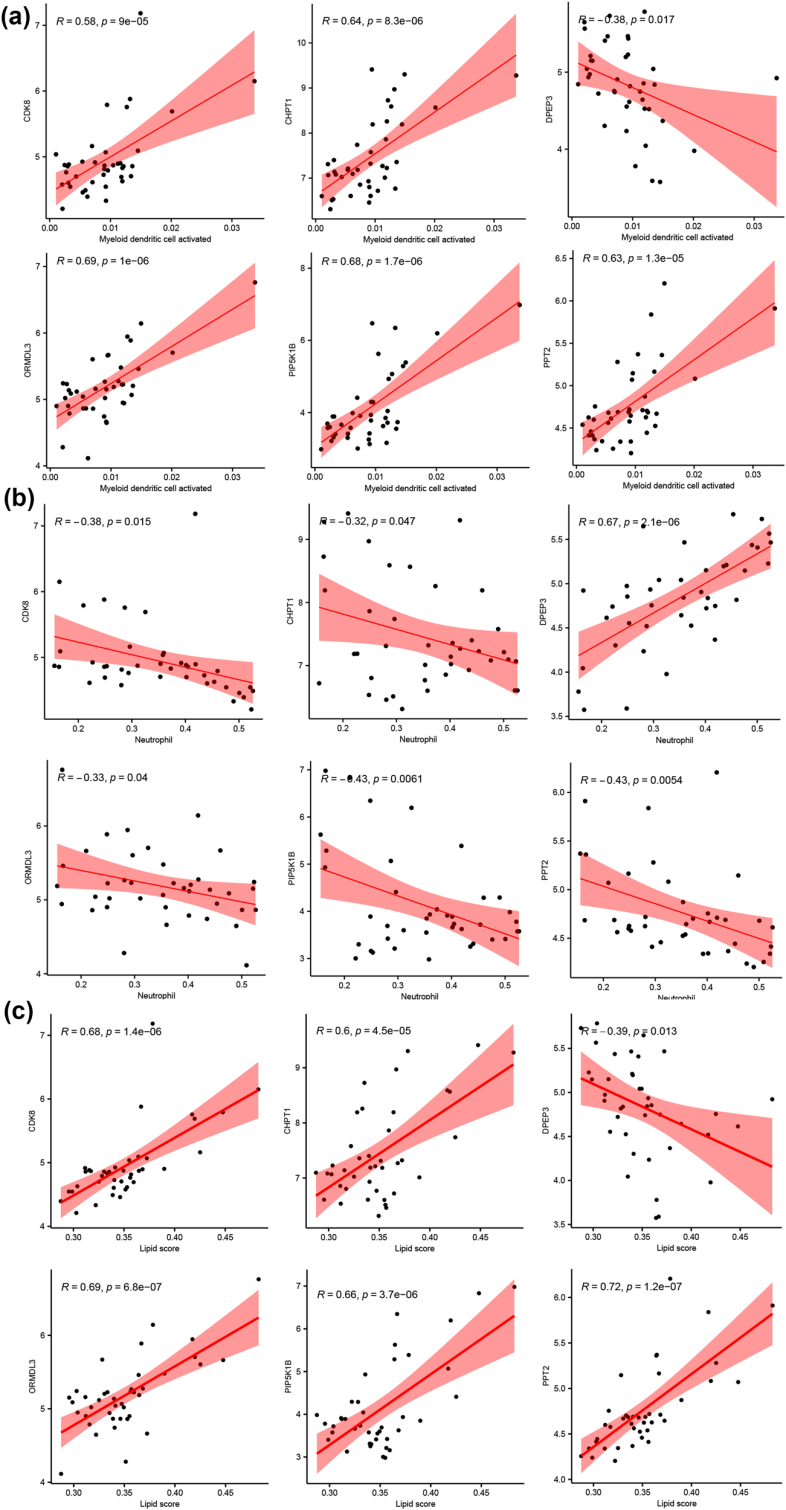
Correlations between expression levels of six genes and myeloid dendritic cell activated (a), neutrophil (b), and lipid score (c).

Finally, the immune cell distributions in the GSE123568 samples were evaluated using CIBERSORT, as shown in Figure 8a. As determined using the Kruskal–Wallis test, two immune cell types, myeloid dendritic cell activated and neutrophil, differed significantly between the two groups (Figure 8b); the frequency of myeloid dendritic cell activated was significantly higher in CRTL than in SONFH (*p* < 0.05), while that of neutrophil showed the opposite pattern (*p* < 0.001). Immune cell frequencies were positively correlated with the expression levels of *CDK8*, *CHPT1*, *ORMDL3 PIP5K1B*, and *PPT2* and *DPEP3* expression was negatively correlated with myeloid dendritic cell activated (Figure 9a). The directions of the correlations between genes and neutrophil and myeloid dendritic cell activated frequencies were opposite (Figure 9b). Additionally, the six genes were significantly correlated with the lipid score (Figure 9c).

## 4 Discussion

Owing to the high disability rate and difficulty in osteonecrotic tissue repair, SONFH has attracted increasing attention [33]. There is recent evidence for the role of abnormal lipid metabolism in SONFH [5,34]. In this study, we performed a multi-step integrative bioinformatics analysis of microarray data to identify lipid metabolism-related DEGs as diagnostic biomarkers of SONFH. Using gene expression profiles in the GSE123568 dataset, a total of 1,724 DEGs (948 downregulated and 776 upregulated) were identified. Additionally, 738 lipid metabolic pathway-related genes were obtained from the MSigDB of GSEA. A module analysis and optimized gene identification revealed six lipid metabolism-related DEGs. The SVM model was used to predict the potential application of biomarkers in SONFH diagnosis, with high AUC values (i.e., 0.957 for the GSE123568 dataset and 0.912 for the GSE74089 dataset), suggesting that this risk classifier has good discriminatory ability between SONFH and CTRL groups. Our results demonstrated that these six optimized lipid metabolism-related DEGs can be used as potential biomarkers for the diagnosis of SONFH.

A total of 18 overlapping lipid metabolism-related, SONFH-related genes were identified. We identified six optimized lipid metabolism-related DEGs among the overlapping DEGs, namely *CDK8*, *CHPT1*, *DPEP3*, *ORMDL3*, *PIP5K1B*, and *PPT2*. Previous studies have indicated that immune cell infiltration and inflammatory pathway activation significantly impact the occurrence and development of femoral head necrosis [15,34,35]. The correlations between immune cells and SONFH have been investigated extensively [35,36]. A previous study has indicated that circRNA-CDK8 is involved in osteogenic differentiation via influencing autophagy and apoptosis in a hypoxic microenvironment [37]. CDK8 regulates *de novo* lipogenesis and lipid accumulation [38] and is related to lipid production, accumulation, and metabolism [38–40]. In addition, CDK8 is closely related to immune cells, including NK cells [41] and activated dendritic cells [42]. Dorighello et al. revealed that CHPT1 contributes to *de novo* phosphatidylcholine synthesis [43]. It may participate in lipid metabolism disorders by regulating glycerophospholipid metabolism in silicosis [44]. DPEP3, a member of the DPEP family, is associated with immune responses and cholesterol clearance in vascular tissues [45,46]. The substrate of DPEP2 is a pro-inflammatory lipid mediator [47]. Lai et al. have shown that DPEP3 expression is positively related to the levels of IL-1α, IL-1β, and IL-17A and may be involved in inflammation [48]. ORMDL3 plays important role in metabolic diseases [49]. Members of the ORMDL family are involved in *de novo* sphingolipid synthesis [50], and ORMDL3 contributes to autoimmune/inflammatory diseases [51]. PIP5Ks and PIP4Ks are two lipid kinases families [52]. PIP5K1B encodes PIP5Kβ and is associated with CD28 stimulation; it participates in T cell activation and lipid raft accumulation [53]. PPT2, a lipase, may play a special role in lysosomal lipid metabolism [54]. These results indicated that the candidate genes may contribute to the development of SONFH via lipid metabolism and immune responses. In addition, the six-gene combination has relatively high reference value for the diagnosis of SONFH.

The study results should be validated in larger clinical samples using bioinformatics approaches. Furthermore, the clinical diagnostic value of the six-gene model should be evaluated in additional *in vitro* and *in vivo* studies.

Several studies have developed prognostic models for SONFH based on DEGs. For instance, a partial least squares model exhibited high predictive accuracy (81.34–91.20%) and AUC (0.804–0.901) when used to distinguish patients with SONFH from controls in a large clinical cohort, suggesting its potential contribution to the diagnosis and prevention of femoral head collapse [55]. Wu et al. conducted an ROC curve analysis based on ten endoplasmic reticulum stress-related genes, finding that all hub genes had AUC values greater than 0.6, confirming the diagnostic potential of these genes for SONFH [56]. Similarly, Liang et al. found 11 hub genes with high diagnostic value in a comparison between SONFH and non-SONFH samples (AUC >  0.9) [57]. In this study, the diagnostic value of six optimized lipid metabolism-related DEGs in SONFH was assessed using the SVM model. An ROC analysis demonstrated good diagnostic performance for SONFH (AUC values for GSE123568 and GSE74089 of 0.957 and 0.912, respectively). These results indicate that the six optimized lipid metabolism-related DEGs possess good diagnostic ability for SONFH.

The study had several limitations. First, the diagnostic efficiency and prognostic value of the critical genes were analyzed and validated solely within the GEO dataset. Second, most of the conclusions were derived from bioinformatics analyses. Therefore, additional experiments are necessary to validate these findings using tumor samples and clinical data. Additionally, *in vivo* and *in vitro* experiments could further elucidate the functions of the critical genes in SONFH.

## 5 Conclusion

In conclusion, this study showed that *CDK8*, *CHPT1*, *DPEP3*, *ORMDL3*, *PIP5K1B*, and *PPT2* may play important roles in the pathogenesis of SONFH and may be novel biomarkers. A model based on the six genes had relatively high auxiliary diagnostic value for SONFH.

## Abbreviations

DEGs: differentially expressed genes
GO: gene ontology
GSEA: Gene Set Enrichment Analysis
KEGG: Kyoto Encyclopedia of Genes and Genomes
NONFH: nontraumatic osteonecrosis of the femoral head
ROC: receiver operating characteristic
SONFH: steroid-induced osteonecrosis of femoral head
SVM: support vector machine
WGCNA: weighed gene co-expression network analysis
